# Transport Properties of Flexible Composite Electrolytes Composed of Li_1.5_Al_0.5_Ti_1.5_(PO_4_)_3_ and a Poly(vinylidene fluoride-*co*-hexafluoropropylene) Gel Containing a Highly Concentrated Li[N(SO_2_CF_3_)_2_]/Sulfolane Electrolyte

**DOI:** 10.1021/acsomega.1c02161

**Published:** 2021-06-09

**Authors:** Ji-young Ock, Miki Fujishiro, Kazuhide Ueno, Izuru Kawamura, Ryoichi Tatara, Kei Hashimoto, Masayoshi Watanabe, Kaoru Dokko

**Affiliations:** † Department of Chemistry and Life Science, Yokohama National University, 79-5 Tokiwadai, Hodogaya-ku, Yokohama 240-8501, Japan; ‡ Advanced Chemical Energy Research Center, Institute of Advanced Sciences, Yokohama National University, 79-5 Tokiwadai, Hodogaya-ku, Yokohama 240-8501, Japan; § Unit of Elements Strategy Initiative for Catalysts & Batteries (ESICB), Kyoto University, Kyoto 615-8510, Japan

## Abstract

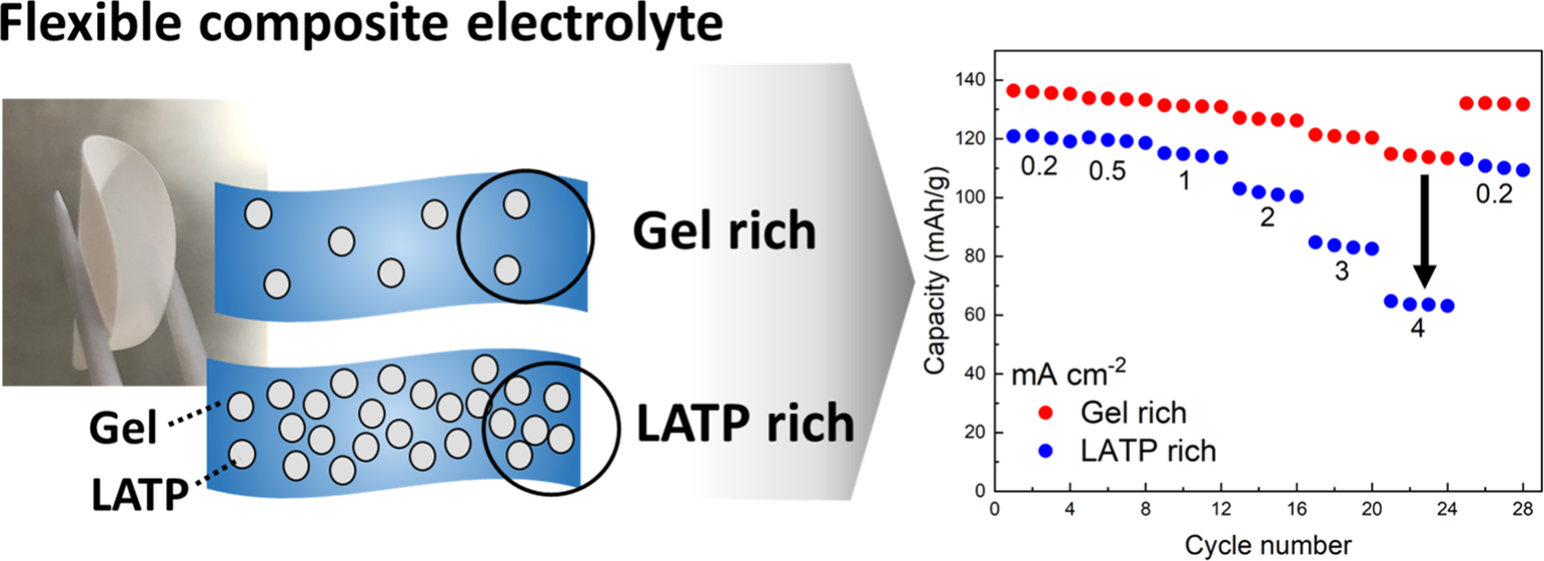

Flexible solid-state electrolyte membranes are beneficial for feasible construction of solid-state batteries. In this study, a flexible composite electrolyte was prepared by combining a Li^+^-ion-conducting solid electrolyte Li_1.5_Al_0.5_Ti_1.5_(PO_4_)_3_ (LATP) and a poly(vinylidene fluoride*-co*-hexafluoropropylene) (PVDF–HFP) gel containing a highly concentrated electrolyte of Li[N(SO_2_CF_3_)_2_] (LiTFSA)/sulfolane using a solution casting method. We successfully demonstrated the operation of Li/LiCoO_2_ cells with the composite electrolyte; however, the rate capability of the cell degraded with increasing LATP content. We investigated the Li-ion transport properties of the composite electrolyte and found that the gel formed a continuous phase in the composite electrolyte and Li-ion conduction mainly occurred in the gel phase. Solid-state ^6^Li magic-angle spinning NMR measurements for LATP treated with the ^6^LiTFSA/sulfolane electrolyte suggested that the Li^+^-ion exchange occurred at the interface between LATP and ^6^LiTFSA/sulfolane. However, the kinetics of Li^+^ transfer at the interface between LATP and the PVDF–HFP gel was relatively slow. The interfacial resistance of LATP/gel was evaluated to be 67 Ω·cm^2^ at 30 °C, and the activation energy for interfacial Li^+^ transfer was 39 kJ mol^–1^. The large interfacial resistance caused the less contribution of LATP particles to the Li-ion conduction in the composite electrolyte.

## Introduction

Li-ion-conducting inorganic solid electrolytes (SEs) have been widely investigated because of their high thermal stability and single-ion conducting properties.^1−3^ Recently, sulfide-based SEs have shown high ionic conductivity on the order of 10^–3^–10^–2^ S cm^–1^ at room temperature, comparable to that of conventional liquid electrolytes, and a Li-ion cell with a sulfide-based SE has been demonstrated to exhibit high rate performance in a wide temperature range.^4^ Sulfide-based SEs react with moisture and require handling and cell assembly in an inert environment.^5^ Oxide-based SEs, such as Li_7_La_3_Zr_2_O_12_ and Li_1+*x*_ Al_*x*_Ti_2–*x*_(PO_4_)_3_ (LATP),^6−9^ have also been investigated because they have higher chemical stability than their sulfide counterparts, although the ionic conductivity of oxide-based SEs is relatively low (∼10^–4^ S cm^–1^) at room temperature.^10^ The high resistance to Li^+^ conduction at the grain boundaries in oxide-based SEs is one of the reasons for the low conductivity.^11^ Many research groups have attempted to develop all-solid-state batteries with a Li metal anode because of the high specific capacity of Li metal. However, most inorganic SEs are thermodynamically unstable against Li metal and form decomposition phases, resulting in high electrode–electrolyte interfacial resistance and deterioration in cell performance.^12−14^ The surface roughness and brittleness of inorganic SEs, which make poor interfacial contact, are other reasons for the high interfacial resistance between the solid electrolyte and the electrode.^15,16^ In addition, there are many challenges in the manufacturing process of solid electrolyte sheets with a large area for use in practical Li-ion batteries.

To address the aforementioned issues in the application of inorganic SEs, composite electrolytes composed of inorganic SEs and polymer electrolytes have been investigated.^17−25^ Li^+^ ion-conducting polymer electrolytes are flexible and can be prepared using a solution casting method. This makes it feasible to realize a close contact between the electrode and the polymer electrolyte. In addition, a polymer electrolyte membrane with a large area can be prepared in a feasible manner. Poly(ethylene oxide) (PEO)-based polymer electrolytes are often used to fabricate SE–polymer composite electrolyte sheets.^26^ PEO exhibits excellent characteristics such as processability, flexibility, and Li^+^ ion solvating properties.^27^ However, PEO-based polymer electrolytes have relatively low ionic conductivity, on the order of 10^–5^ S cm^–1^ at room temperature, a low Li-ion transference number (*t*_Li+_) of ∼0.2, and low oxidative stability (<4 V).^28,29^ Low *t*_Li+_ causes concentration polarization when relatively high current density is applied to a polymer electrolyte, which limits the rate capability of Li polymer batteries. Poly(vinylidene fluoride-*co*-hexafluoropropylene) (PVDF–HFP)-based polymer gel electrolytes have been considered another option of flexible solid electrolytes.^30^ PVDF–HFP-based gel electrolytes containing an organic liquid electrolyte exhibit ionic conductivity on the order of ∼10^–3^ S cm^–1^ and high electrochemical stability derived from the liquid electrolyte, while a low Li-ion transference number still poses a challenge in gel electrolytes.

In this work, we prepared a polymer gel electrolyte composed of PVDF–HFP and a highly concentrated Li salt/sulfolane electrolyte. Composite electrolyte membranes comprising an SE, Li_1.5_Al_0.5_Ti_1.5_(PO_4_)_3_ (LATP), and a gel electrolyte were also fabricated. PVDF–HFP exhibits a relatively good mechanical strength and is electrochemically stable against both reduction and oxidation. This polymer can also support a relatively large amount (∼80 wt %) of a liquid electrolyte in the matrix. We previously reported that sulfolane-based highly concentrated Li salt electrolytes exhibit high *t*_Li+_ (0.6–0.8) and that Li batteries can be operated at a current density of ∼2 mA cm^–2^, regardless of the relatively low ionic conductivity (0.3–0.5 mS cm^–1^) at room temperature.^31,32^ In this work, we applied composite electrolytes to Li/LiCoO_2_ cells. In addition, we characterized the mechanical properties and Li^+^ transport properties of the composite electrolyte membranes and found that the interfacial resistance of the LATP/PVDF–HFP gel significantly affects the Li^+^ transport through the membranes.

## Experimental Section

### Synthesis of Li_1.5_Al_0.5_Ti_1.5_(PO_4_)_3_ (LATP)

LATP was prepared using a sol–gel method.^33−36^ Al(OC_4_H_9_)_3_ (97%, Sigma-Aldrich) and Ti(OC_4_H_9_)_4_ (97%, Sigma-Aldrich) were dissolved in *n*-C_4_H_9_OH (99%, Sigma-Aldrich). CH_3_COOLi (98%, Sigma-Aldrich) and NH_4_H_2_PO_4_ (99%, Sigma-Aldrich) were dissolved in purified H_2_O. The two solutions were homogeneously mixed at 60 °C for 2 h to prepare the Li–Al–Ti–(PO_4_) sol. The molar ratio of CH_3_COOLi:Al(OC_4_H_9_)_3_/Ti(OC_4_H_9_)_4_/NH_4_H_2_PO_4_/*n*-C_4_H_9_OH/H_2_O was 1.5:0.5:1.5:3:50:800. The prepared sol was dried at 100 °C, and the obtained powder was calcined at 500 °C for 4 h in air to obtain an amorphous LATP powder. The ground powder was further heat-treated at 950 °C for 12 h to crystallize the LATP powder. An LATP pellet was prepared as follows: the amorphous LATP was pressurized in a 13 mm diameter die at 350 MPa, followed by calcination at 950 °C for 12 h. Both the sides of the obtained LATP pellet were coated with Au using a sputtering method for ionic conductivity measurements.

### Preparation of Composite Membranes

Purified sulfolane (SL) was purchased from Kishida Chemical and used as received. LiTFSA was supplied by Solvay, Japan. LiTFSA and SL were mixed in a 1:2 molar ratio in an Ar-filled glovebox to prepare a highly concentrated liquid electrolyte. This liquid electrolyte is hereinafter abbreviated as [Li(SL)_2_][TFSA]. The composite membranes composed of LATP and the PVDF–HFP gel were prepared in a dry chamber (dew point: −60 °C). PVDF–HFP (Kynar Flex 2801, Arkema) and [Li(SL)_2_][TFSA] were dissolved in anhydrous acetone (99%, Sigma-Aldrich). The weight ratio of PVDF–HFP/[Li(SL)_2_][TFSA] was 30:70. This polymer solution was mixed with the LATP powder, vigorously stirred for 2 h, poured into a glass dish, and dried to obtain a composite membrane. The membrane was dried overnight at room temperature and further dried under vacuum at 60 °C overnight to completely evaporate acetone. The thickness of the obtained membranes was in the range of 60–110 μm. Hereinafter, the composition of the electrolyte is described as Gel*x*-LATP*y* based on the weight percentages of the PVDF–HFP gel (*x*) and LATP (*y*). The composite electrolytes prepared at *y* > 70 were very fragile and did not form a self-standing membrane and were therefore excluded from subsequent studies.

### Characterization of the Composite Electrolyte

The composite membrane was cut into dumbbell shapes, and the tensile properties were analyzed using a Shimadzu EZ-LX at a cross-head speed of 10 mm min^–1^. The Young’s modulus and fracture energy of the composite electrolytes were evaluated from the slope of the stress–strain curve (0.03–0.08 N) and the area under the stress–strain curves, respectively. The morphologies of the composite electrolytes were observed using a field emission scanning electron microscope (FE-SEM, SU8010, Hitachi). The ionic conductivity of the electrolyte was evaluated as follows: the composite membrane was punched into a circular shape and placed between two polished stainless steel (SUS) disk electrodes. The ionic conductivity was measured using a complex impedance analyzer (Hewlett-Packard 4192A) at frequencies ranging from 13 MHz to 5 Hz with a voltage amplitude of 50 mV. To analyze the interfacial resistance between LATP and the PVDF–HFP gel, we used an LATP plate (LiCGC, Ohara Inc.), and a symmetric cell of [SUS/gel/LATP plate/gel/SUS] was assembled in an Ar-filled glovebox. The impedance of the three-layer electrolyte of the gel/LATP plate/gel was measured using an impedance analyzer (Biologic, VMP3) in a frequency range of 1 MHz to 100 mHz with a voltage amplitude of 10 mV.

### Battery Test

Battery-grade LiCoO_2_ (AGC Seimi Chemical) and acetylene black (AB, DENKA) were used as received. LiCoO_2_, AB, and PVDF–HFP (Kynar Flex 2801) were mixed in a weight ratio of 85:9:6 in a mortar and dispersed in *N*-methylpyrrolidone to obtain a slurry. The slurry was then spread on an Al foil with a doctor blade and dried at 80 °C for over 2 h. The cathode sheet was cut into a circle (13.82 mm diameter) and dried under vacuum at 80 °C for over 2 h. The thickness of the cathode sheet on the Al foil was ca. 30 μm, and the typical mass loading of LiCoO_2_ was 2.7 mg cm^–2^. The cathode sheet, composite electrolyte, and Li metal anode were encapsulated in a 2032-type coin cell. Thirty microliters of [Li(SL)_2_][TFSA] was added between the cathode and the composite electrolyte. The cell was assembled in a glovebox and aged at 60 °C for one day before testing. Galvanostatic charge–discharge measurements were performed using an automatic charge–discharge instrument (HJ1001SD8, Hokuto Denko) at 60 °C.

## Results and Discussion

**Figure 1:**
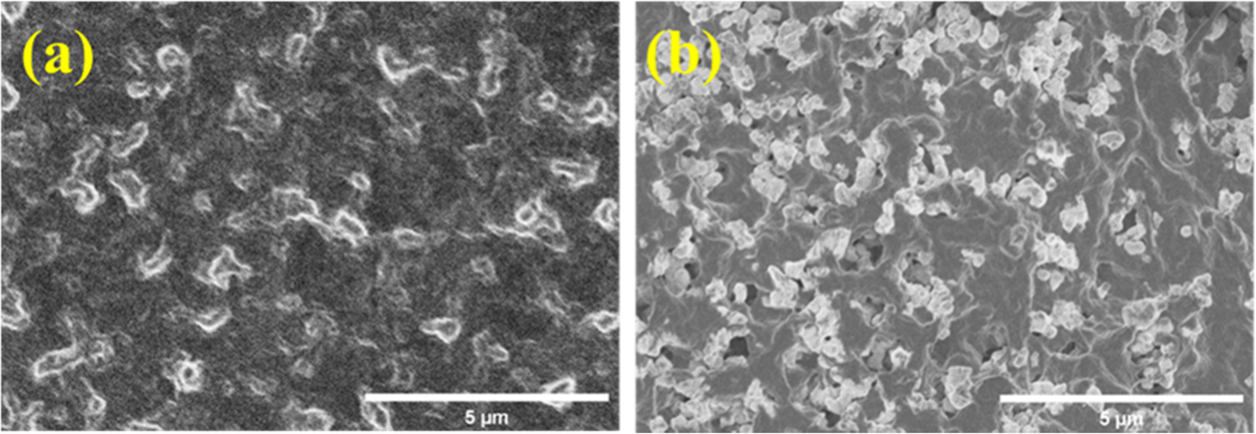
FE-SEM images of the surfaces of composite electrolytes of (a) Gel100 (without LATP) and (b) Gel40-LATP60.

The ionic conductivity of the gel electrolyte composed of 30 wt % PVDF–HFP and 70 wt % [Li(SL)_2_][TFSA] was 0.146 mS cm^–1^ at 30 °C, which is slightly lower than that of the liquid [Li(SL)_2_][TFSA] electrolyte (0.423 mS cm^–1^ at 30 °C). The particle size of the LATP powder prepared using the sol–gel method was <2 μm (Figure S1). LATP contained a small amount of AlPO_4_ as an impurity (Figure S2), possibly derived from the thermal decomposition of LATP during calcination at high temperatures.^37^ Although the impurity existed in the sample, the sintered LATP pellet exhibited an ionic conductivity of 1.31 × 10^–5^ S cm^–1^ at 30 °C, which is comparable to the reported values.^33,36^ We prepared composite electrolytes composed of the PVDF–HFP gel and the LATP powder via a solution casting process. Figure 1 shows the FE-SEM images of the composite electrolytes. For the Gel100 membrane (without LATP), no pores can be observed over the surface, indicating that the membrane was uniformly formed during the evaporation of the casting solvent.^38^ However, small pores can be observed in Gel70-LATP30 (not shown) and Gel40-LATP60 (Figure 1b). The pores might have been created during the evaporation of the solvent (acetone) between the LATP particles.

**Figure 2:**
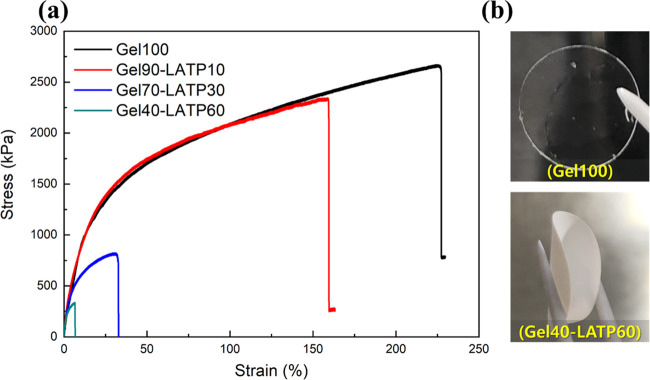
(a) Stress–strain curves of the composite electrolytes with different LATP compositions measured at room temperature. (b) Images of Gel100 and Gel40-LATP60 membranes.

Tensile tests were performed to evaluate the mechanical strength of the membranes. Figure 2a shows the stress–strain curves of the composite membranes, and the mechanical properties are summarized in Table S1. Gel100 showed remarkable deformation properties, and the fracture strain was 224% with a tensile strength of 2.67 MPa. With an increase in the amount of LATP, the fracture strain gradually decreased. However, notably, even at a high content of LATP (Gel40-LATP60), the composite electrolyte remained flexible, as shown in Figure 2b.

**Figure 3:**
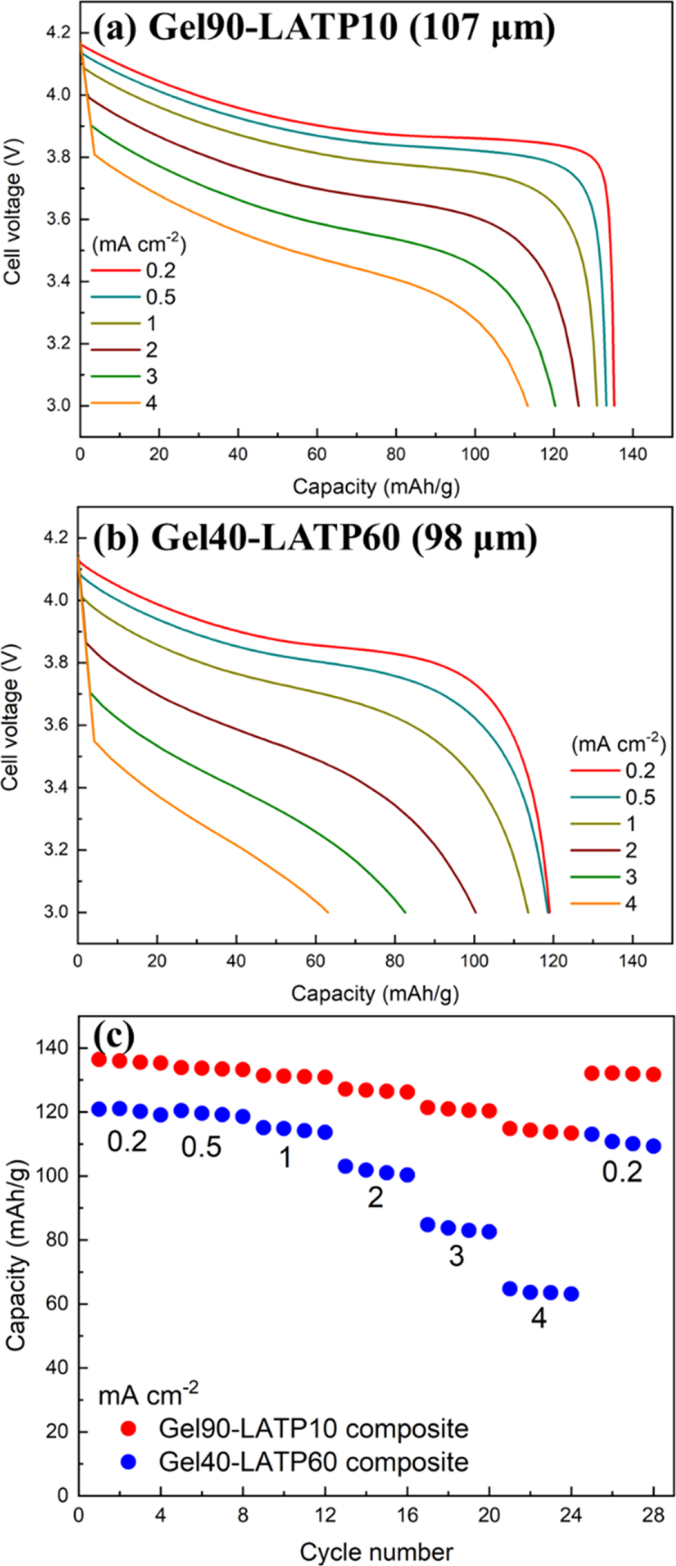
Discharge curves of [Li/composite electrolyte/LiCoO_2_] cells with (a) Gel90-LATP10 and (b) Gel40-LATP60 composite electrolytes measured at 60 °C. (c) Discharge capacities of the cells measured at various current densities. The cells were charged up to 4.2 V at a current density of 0.2 mA cm^–2^ prior to each discharge.

Li/LiCoO_2_ cells were assembled with composite electrolytes (thickness: ca. 100 μm), and charge–discharge tests were conducted at 60 °C. Figure 3 shows the discharge curves of the cells with Gel90-LATP10 and Gel40-LATP60 measured at various current densities. At a low current density of 0.2 mA cm^–2^, the cells exhibited discharge capacities in the range of 120–140 mAh g^–1^, which is close to the theoretical capacity (137 mAh g^–1^) of the redox reaction of LiCoO_2_/Li_0.5_CoO_2_.^39^ LATP is known to react with Li metal, and Ti^4+^ in LATP is reduced to Ti^3+^ through the reaction. However, Li/LiCoO_2_ could be operated successfully with the composite electrolytes. Probably, the LATP particles in contact with Li metal were reduced; however, the reduction reaction of LATP did not propagate inside the composite electrolytes due to the low electronic conductivity of LATP. In addition, the gel electrolyte between the LATP particles might inhibit the propagation of the reduction reaction. As shown in Figure 3, with increasing current density, the discharge voltage decreases because of the IR drop in the electrolyte membrane and the overvoltage for the electrochemical reactions in the cells. Apparently, the discharge voltage of the cell with Gel40-LATP60 was lower than that of the cell with Gel90-LATP10 at high current densities. In addition, the discharge capacity of the cell with Gel40-LATP60 was lower than that of the cell with Gel90-LATP10. The cells with Gel90-LATP10 and Gel40-LATP60 showed discharge capacities of 113 and 63 mAh g^–1^, respectively, at 4 mA cm^–2^. These results suggest that the internal resistance of the cell with Gel40-LATP60 was higher than that of the cell with Gel90-LATP10.

**Figure 4:**
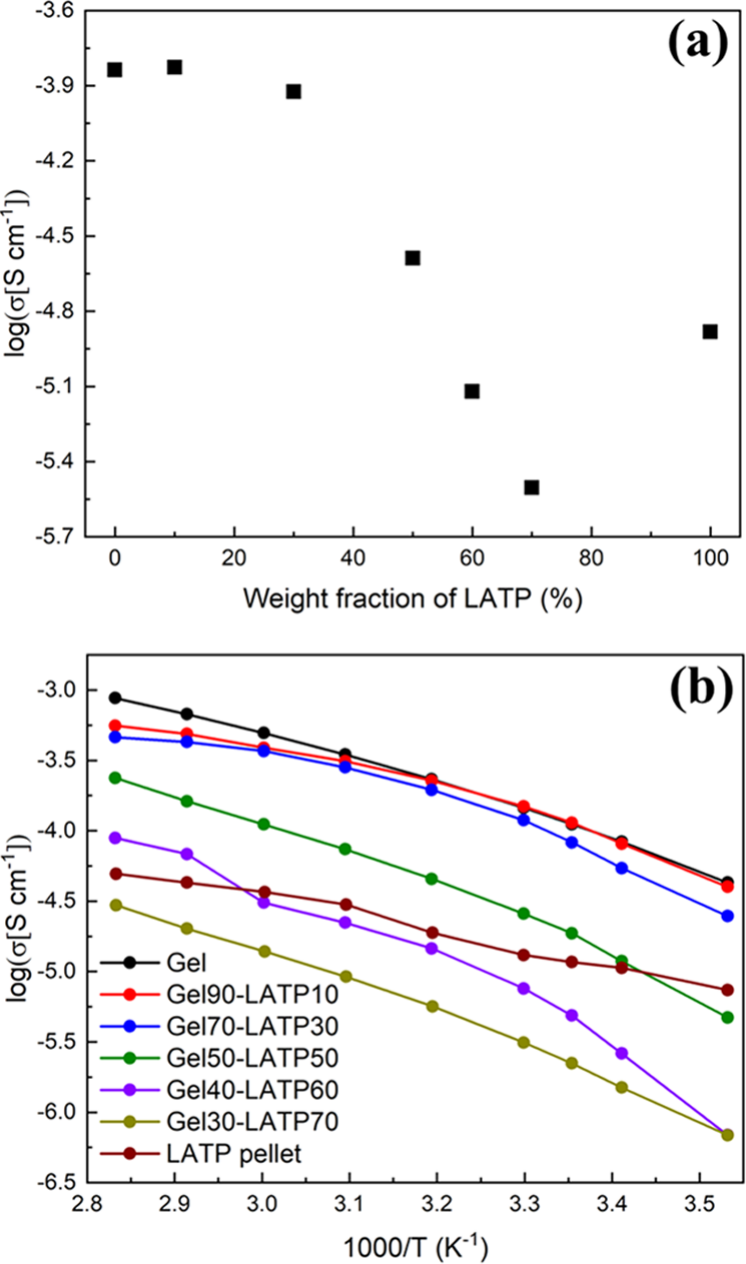
(a) Ionic conductivity of the composite electrolytes as a function of the LATP content at 30 °C. (b) Arrhenius plots of the conductivity of composite electrolytes.

The ionic conductivity of the composite electrolyte was measured to determine the origin of the internal resistance of the cell. Figure 4a shows the ionic conductivity of the composite electrolytes with various LATP contents at 30 °C. The ionic conductivity of the composite electrolyte decreases with increasing LATP fraction and became as low as 3.13 × 10^–6^ S cm^–1^ in Gel30-LATP70, which is lower than that of the LATP pellet (1.31 × 10^–5^ S cm^–1^). Figure 4b shows the Arrhenius plots of the conductivity of the electrolytes. The Arrhenius plots of the conductivities of composite electrolytes showed convex-curved profiles, which are common behaviors of ionic conduction in organic electrolytes and can be expressed by the Vogel–Fulcher–Tamman (VFT) equation.^40^ Apparently, the activation energy for ionic conduction in each composite electrolyte is similar to that of the gel electrolyte (without LATP). These suggest that ionic conduction mainly occurs in the gel phase. This indicates that the LATP particles in the composite electrolyte hardly contribute to ionic conduction. In the composite electrolytes, the LATP particles might be scarcely connected to each other (Figure 1) and do not form a continuous phase. Indeed, the Young’s modulus of the composite electrolyte is largely independent of the LATP content (Figure 2 and Table S1), suggesting that the elastic property of the composite electrolyte is mainly due to the gel phase. In other words, the gel forms a continuous phase in the composite electrolyte, and the LATP particles are dispersed in the gel matrix.

The lower contribution of LATP to ionic conduction in the composite electrolyte implies that Li^+^ cannot pass through the LATP particles. If there is Li^+^-ion exchange between the gel and LATP, LATP may contribute to ion conduction to some extent. To examine whether Li^+^-ion exchange occurs, we simply mixed the LATP powder and the liquid electrolyte of [^6^Li(SL)_2_][TFSA] for 48 h. Subsequently, solid-state ^6^Li magic-angle spinning (MAS) NMR measurements were performed on the LATP powder. Based on the natural abundance of the ^6^Li (7.59%) ion, ^6^Li exchanged between the [^6^Li(SL)_2_][TFSA] electrolyte and LATP can be assessed quantitatively. Figure S3 (Supporting Information) shows the ^6^Li NMR spectra for the pristine LATP powder and the LATP powder treated with the liquid electrolyte of [^6^Li(SL)_2_][TFSA]. The ^6^Li signal for the pristine LATP was observed at a chemical shift of −1.15 ppm, which is consistent with the reported value.^41^ The intensity of the ^6^Li signal was significantly increased after mixing with the [^6^Li(SL)_2_][TFSA] electrolyte, suggesting that Li^+^ exchange occurred at the interface between LATP and [Li(SL)_2_][TFSA] and ^6^Li^+^ diffused into the bulk of the LATP particles. Regardless of the Li^+^ exchange, the LATP particles hardly contribute to ionic conduction in the composite electrolyte. A possible hypothesis is the slow kinetics of Li^+^-ion exchange at the LATP/gel interface. If the interfacial Li-ion exchange is relatively slow, Li ions mainly migrate within the continuous gel phase and do not often pass through the LATP/gel interface in the composite electrolyte.

**Figure 5:**
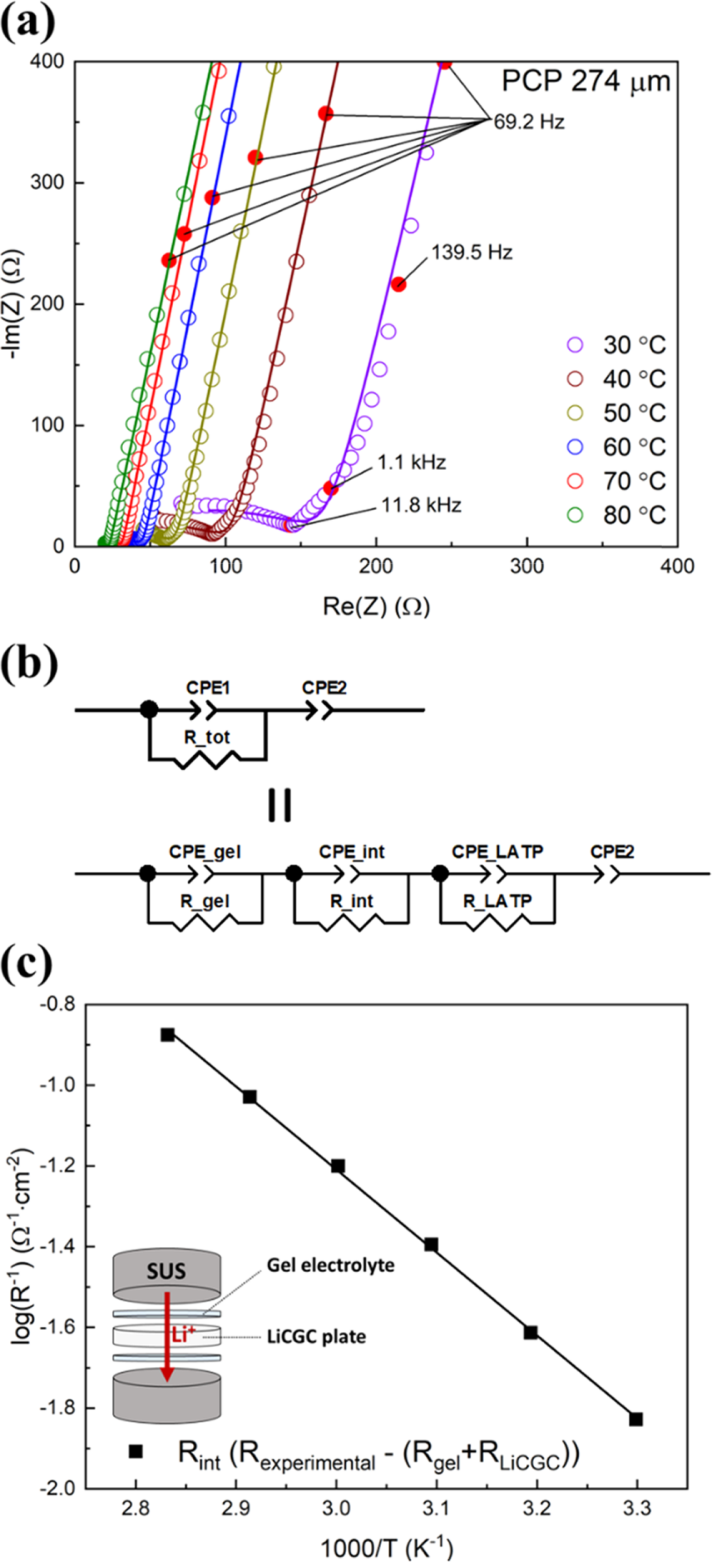
(a) Nyquist plots of an SUS/gel/LiCGC/gel/SUS cell measured at various temperatures. The area of each gel electrolyte is 2 cm^2^, and the total thickness of the two gel electrolytes is 124 μm. The area of the LiCGC plate is 2 cm^2^ with a thickness of 150 μm. (b) Equivalent circuit model of the SUS/gel/SE/gel/SUS cell. Constant phase elements (CPEs) are used instead of capacitances to fit the impedance spectra. (c) Arrhenius plot of 1/*R*_int_. *R*_int_ is normalized using the contact area (2 cm^2^) of the LiCGC/gel electrolyte and divided by 2 (number of interfaces).

To investigate the rate of Li^+^ exchange at the interface between the gel electrolyte and LATP, a symmetric cell of [SUS/gel/LATP plate/gel/SUS] was assembled using an LATP plate (LiCGC, Ohara Inc.), and AC impedance measurements were conducted. LiCGC is a commercially available LATP plate and has a relatively high ionic conductivity of 1.16 × 10^–4^ S cm^–1^ at 30 °C (Figure S4). Figure 5a shows the Nyquist plots of the symmetric cell measured at various temperatures. A depressed semicircle was observed in the high-frequency region (>10 kHz), and a sloping line appeared at frequencies lower than 10 kHz. Figure 5b shows the equivalent circuit model for the SUS/Gel/SE/Gel/SUS cell. The depressed semicircle in the high-frequency region is assumed to originate from the resistances of the LiCGC plate (*R*_LATP_), the gel electrolyte (*R*_gel_), and the interfacial resistance between LiCGC and the PVDF–HFP gel (*R*_int_). The diameter of the semicircle, *R*_tot_, is the sum of *R*_LATP_, *R*_gel_, and *R*_int_. We could not distinguish *R*_LATP_, *R*_gel_, and *R*_int_ because the time constants of the interfacial Li^+^ transfer process at LATP/gel and ion conduction in LATP and the PVDF–HFP gel were similar. Therefore, AC impedance measurements were conducted on the LICGC plate and the gel electrolyte sheet separately, and their resistivities were evaluated (Figure S4). From the resistivities, the resistances *R*_LATP_ and *R*_gel_ in the three-layer gel/SE/gel electrolyte cell were calculated, and the interfacial resistance *R*_int_ was estimated as follows: *R*_int_ = *R*_tot_ – *R*_LATP_ – *R*_gel_. Figure 5c shows the Arrhenius plot of 1/*R*_int_, where the Li-ion transfer rate at the interface is proportional to 1/*R*_int_. The *R*_int_ value is the sum of the two interfacial resistances of gel/LICGC/gel, and the normalized interfacial impedance of a single interface of LICGC/gel was 67 Ω cm^2^ at 30 °C. The activation energy for the charge transfer (i.e., Li-ion transfer) at the interface of the LICGC/gel was estimated to be 39 kJ mol^–1^, whereas the activation energies of ion conduction in LiCGC and the PVDF–HFP gel were 36 and 32 kJ mol^–1^, respectively. As discussed previously, Li^+^ conduction in the LATP gel composite electrolyte mainly occurs in the gel phase. The passage through the interface of LATP/gel is unfavorable for Li^+^-ion conduction in the composite electrolyte because of the interfacial resistance of LATP/gel and the activation barrier for interfacial Li-ion transfer. Although we cannot conclude what determines the Li-ion transfer rate at the LATP/gel interface currently, the interaction between Li^+^ and ligands (solvent and anion) in the gel electrolyte and the interaction between Li^+^ and anions in LATP would certainly affect the Li-ion transfer process because the environment of Li^+^ in the gel electrolyte and LATP should be significantly different. To achieve a higher Li-ion transfer rate (or lower *R*_int_), further investigations are required.

## Conclusions

In this work, flexible composite electrolytes comprising the LATP powder and the PVDF–HFP gel containing [Li(SL)_2_][TFSA] were prepared using a solution casting method. The prepared composite electrolytes possessed sufficient mechanical strength as separators applicable to lithium batteries. Li/LiCoO_2_ cells could be operated successfully with a composite electrolyte; however, the rate capability of the cell degraded with increasing LATP content in the composite electrolyte. The ionic conductivity of the composite electrolyte decreased with increasing LATP content. In the composite electrolytes, the gel formed a continuous phase, and Li-ion conduction mainly occurred in the gel phase. The LATP particles contributed less to Li-ion conduction in the composite electrolytes, which was attributed to the resistance to Li^+^ transfer at the interface between LATP and the PVDF–HFP gel. The interfacial resistance of LATP/gel was 67 Ω·cm^2^ at 30 °C, and the activation energy for interfacial Li^+^ transfer was estimated to be 39 kJ mol^–1^. The large interfacial resistance caused the less contribution of LATP particles to the Li-ion conduction in the composite electrolytes.

## Supporting Information Available

FE-SEM images of the LATP powder (Figure S1); X-ray diffraction (XRD) patterns of synthesized LATP (Figure S2); Young’s modulus, tensile strength, fracture strain, and fracture energy of composite electrolytes (Table S1); ^6^Li NMR spectra (Figures S3); and Nyquist plots and ionic conductivity of the LiCGC plate (Figure S4) (PDF)
