# Supplementary material for: Transport Properties of Flexible Composite Electrolytes Composed of Li1.5Al0.5Ti1.5(PO4)3 and a Poly(vinylidene fluoride-co-hexafluoropropylene) Gel Containing a Highly Concentrated Li[N(SO2CF3)2]/Sulfolane Electrolyte
Source: ACS Omega. 2021 Jun 9;6(24):16187–93. doi: 10.1021/acsomega.1c02161 (PMC8223415; doi:10.1021/acsomega.1c02161)
Supplement: Supplementary file 1 — ao1c02161_si_001.pdf [file ao1c02161_si_001.pdf]

Supporting Information

Transport Properties of Flexible Composite Electrolytes Composed of $Li_{1.5}Al_{0.5}Ti_{1.5}(PO_4)_3$ and Poly(vinylidene fluoride-*co*-hexafluoropropylene) Gel Containing a Highly Concentrated $Li[N(SO_2CF_3)_2]$/Sulfolane Electrolyte

Ji-young Ock,[†] Miki Fujishiro,[†] Kazuhide Ueno,[†,‡] Izuru Kawamura,[†] Ryoichi Tatara,[†] Kei Hashimoto,[‡] Masayoshi Watanabe,[‡] and Kaoru Dokko [†,‡,§,*]

[†]Department of Chemistry and Life Science, Yokohama National University, 79-5 Tokiwadai, Hodogaya-ku, Yokohama 240-8501, Japan

[‡]Advanced Chemical Energy Research Center, Institute of Advanced Sciences, Yokohama National University, 79-5 Tokiwadai, Hodogaya-ku, Yokohama 240-8501, Japan

[§]Unit of Elements Strategy Initiative for Catalysts & Batteries (ESICB), Kyoto University, Kyoto 615-8510, Japan

*Corresponding Author: dokko-kaoru-js@ynu.ac.jp

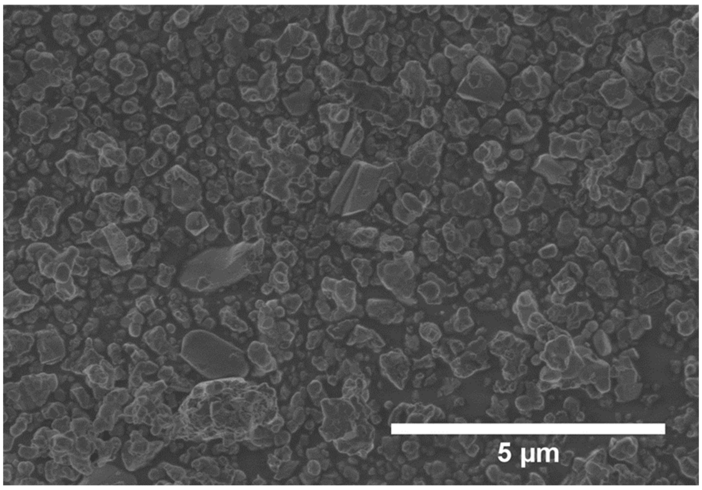

Figure S1. FE-SEM images of LATP powder heat treated at 950 ℃ for 12 h.

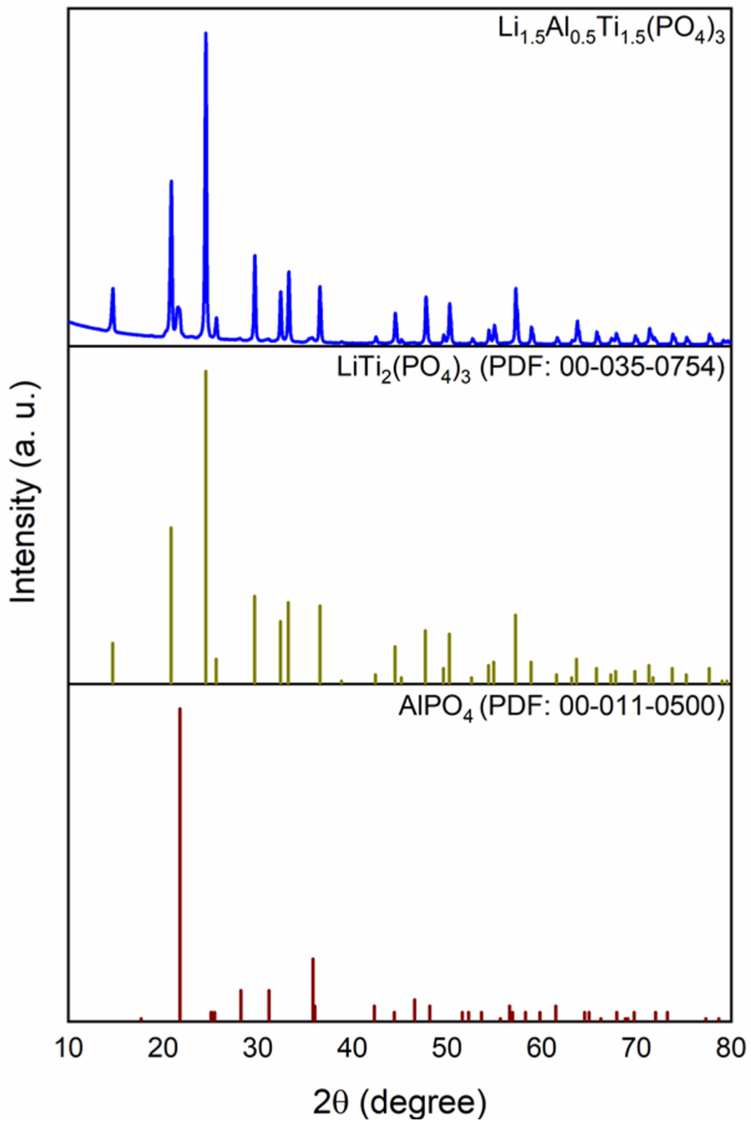

Figure S2. XRD patterns of synthesized LATP particles.

Table S1. Young's modulus, tensile strength, fracture strain, and fracture energy of composite electrolytes with different amounts of LATP particles.

[Gel]-[LATP] / Weight %	pvdf-*co*-hfp / Weight %	Young's modulus / MPa (0.03–0.08 N)	Tensile strength / MPa	Fracture strain / %	Fracture energy / kJ m^{-3}
Gel100	30	8.78	2.67	224	4528
Gel90-LATP10	27	10.44	2.34	159	2886
Gel70-LATP30	21	10.27	0.82	30	188
Gel40-LATP60	12	7.06	0.34	6	16

Solid-state ^{6}Li MAS–NMR measurements

$^6LiN(SO_2CF_3)_2$ (LiTFSA) was synthesized by neutralization of $HN(SO_2CF_3)_2$ (HTFSA, 99%, Kanto Chemical) and $^6Li_2CO_3$ (95%, Sigma-Aldrich). $^6Li_2CO_3$ was mixed with HTFSA in a 1:2 molar ratio in water and stirred overnight. After neutralization, the water was evaporated using a rotary evaporator to obtain ^{6}LiTFSA. ^{6}LiTFSA was further dried under vacuum at 120 °C for over 24 h. The obtained ^{6}LiTFSA was stored in an Ar-filled glove box (VAC, [H_2O] <0.5 ppm). Sulfolane（SL）and ^{6}LiTFSA were mixed in a 2:1 molar ratio and stirred overnight at 60 °C to obtain a homogeneous liquid of [$^6Li(SL)_2$][TFSA].

The Li^+ ion exchange between [$^6Li(SL)_2$][TFSA] and LATP powder was examined. The LATP powder was immersed in [$^6Li(SL)_2$][TFSA] and stirred at 30 °C for over 48 h. Subsequently, the LATP was washed with tetrahydrofuran (99%, Wako Chemical) several times to remove [$^6Li(SL)_2$][TFSA]. The Li^+-exchanged LATP powder was dried under vacuum at 50 °C overnight and then transferred to a 4.0 mm o.d. zirconia-type NMR tube.

Solid-state ^{6}Li magic-angle spinning (MAS) NMR measurements were performed on a Bruker 600 MHz AVANCE NMR spectrometer operated at a ^{6}Li resonance frequency of 88.31 MHz. ^{6}Li NMR spectra using a 90° excitation pulse of 3.6 μs and a repetition time of 100 s were acquired at an MAS frequency of 13 kHz at room temperature. The ^{6}Li chemical shift was externally referenced to 1 M LiCl aqueous solution at 0.0 ppm.

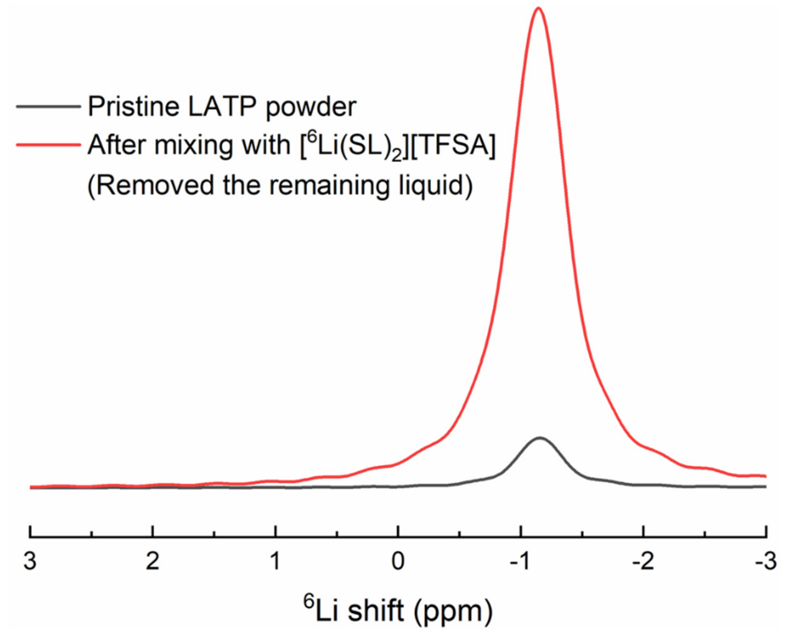

Figure S3. ^{6}Li NMR peak comparison of pristine LATP powder (black) and that mixed with [$^6Li(SL)_2$][TFSA] at 30 °C for two days (red).

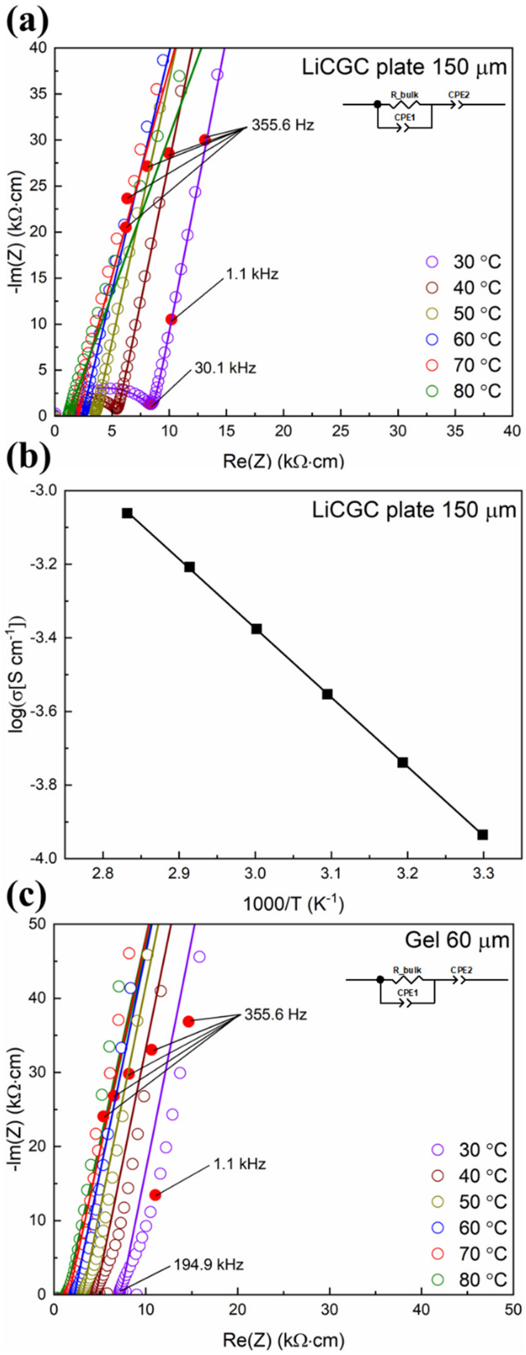

Figure S4. (a) Nyquist plots and (b) ionic conductivity of the LiCGC plate measured at various temperatures. Au is coated on both sides of the LATP plate using a sputtering method, and the AC impedance is measured. (c) Nyquist plots of PVDF–HFP gel measured at various temperatures.
